# *Devosia nitraria* sp. nov., a novel species isolated from the roots of *Nitraria sibirica* in China

**DOI:** 10.1007/s10482-017-0901-z

**Published:** 2017-06-24

**Authors:** Lin Xu, Yong Zhang, Nicholas Read, Shuangjiang Liu, Ville-Petri Friman

**Affiliations:** 1 Key Laboratory of Hexi Corridor Resources Utilization, Hexi University, Zhangye, 734000 Gansu China; 2 Institute of Agricultural and Biological Technology, Hexi University, Zhangye, 734000 Gansu China; 3 Department of Biology, University of York, York, YO10 5DD UK; 4 State Key Laboratory of Microbial Resources, Institute of Microbiology, Chinese Academy of Sciences, Beijing, 100101 People’s Republic of China

**Keywords:** *Devosia*, Novel species, *Nitraria sibirica*

## Abstract

An aerobic, Gram-stain negative, short rod-shaped and motile strain, 36-5-1^T^, was isolated from the roots of *Nitraria sibirica* in Zhangye city, Gansu province, north-west of China. Phylogenetic analysis based on the 16S rRNA gene sequence and two housekeeping genes (*glnA* and *atpD*) indicated that the strain represents a novel species closely related to the *Devosia*, *Rhizobium* and *Devosia* genera with 98.3, 96.2 and 91.1% similarities, respectively. The strain 36-5-1^T^ contained Q-10 as the predominant ubiquinone and 16:0 (36.8%) as the major fatty acid; a large amount of unidentified glycolipid, diphosphatidylglycerol, phosphatidylglycerol and a small amount of unidentified polar lipids were present as polar lipids. In addition, the G+C content of the genomic DNA was 61.7 mol% and the DNA–DNA hybridization with type strains *Devosia geojensis* BD-c194^T^ and *Devosia pacifica* NH131^T^ 44.1 ± 1.1 and 40.2 ± 1.7, respectively. Based on chemotaxonomic data and molecular properties, strain 36-5-1^T^ represents a novel species within the genus *Devosia*, for which the name *Devosia nitraria* sp. nov. is proposed. The type strain is 36-5-1^T^ (=CGMCC1.15704^T^=NBRC112416^T)^.

**Electronic supplementary material:**

The online version of this article (doi:10.1007/s10482-017-0901-z) contains supplementary material, which is available to authorized users.

## Introduction

The genus *Devosia* was first described by Nakagawa et al. (1996) with the reclassification of ‘*Pseudomonas riboflavina*’ (Foster 1944) as *Devosia riboflavina*. Members of this genus are characterized as Gram-stain negative, aerobic, oval or rod-shaped and non-spore forming bacteria. The genus is currently comprised of twenty one validly named species (http://www.bacterio.net/devosia.html).

*Nitraria* is a genus belonging to the family *Zygophyllaceae*. The genus consists of 15 species of deciduous shrubs. These shrubs are widely distributed in the Middle East, central Asia and in north-west region of China and they have special physiological properties in terms of drought and salt resistance. These properties have been shown to prevent soil desertification, by alleviating both the soil salinity and alkalinity (Zhao and Fan 2002), and consequently, the plants have significant ecological value. Five of the species are indigenous to northwestern China. Of the five, *Nitraria sibirica* Pall. is the most common. The fruits of *N. sibirica* Pall. are used in some traditional medicines and are particularly recommended for the treatment of hypertension (Liu 1999). Several studies on *Nitraria* metabolites have shown obvious antihypertensive properties due to the regulation of digestion and spleen functioning (Tulyaganov and Allaberdiev 2001, 2003; Hadj Salem et al. 2011; Suo and Wang 2010). As yet, relatively little is known about the bacterial endophytes of *Nitraria*. Here we describe a novel bacterial endophyte of *Nitraria*, designated as 36-5-1^T^. A polyphasic approach was carried out to classify the bacterium, and according to our current analysis, the strain 36-5-1^T^ likely belongs to the genus *Devosia*.

## Materials and methods

### Strains and culture conditions

A root sample was collected from *N. sibirica* grown in the sand soil of Hexi Corridor to isolate bacterial strains (GPS location: 39°16′N 100°18′E, 1398 m). Endophytes were routinely cultured on either YMA (Vincent 1970) or LB (Bertani 1951) medium at 28 °C and deposited as described by Xu et al. (2016). After primary analysis and sequencing of 16S rRNA, R2A agar (Reasoner and Geldreich 1985) was used to culture the strain.

### Determination of 16S rRNA, recA and atpD gene sequences

The extraction of genomic DNA and PCR amplification of the 16S rRNA gene were conducted as described by Terefework et al. (2001). Amplification of the 16S rRNA gene was performed with two universal primers, F’-27(5′-AGAGTTTGATCCTGGCTCAGAACGAACGCT-3′) and R’-1492 (5′-TACGGCTACCTTGTTACGACTTCACCCC-3′). Amplification of *glnA* and *atpD* genes were performed as described by Gaunt et al. (2001) and Turner and Young (2000), respectively. The gene sequences were compared with a BLAST search of the nucleotide database of the National Center for Biotechnology Information (Altschul et al. 1990) and EzTaxon server 2.1 (http://www.ezbiocloud.net/; Yoon et al. 2016). Sequences were aligned using the CLUSTAL_W software (Thompson et al. 1994). Phylogenetic trees were then generated using neighbour-joining methods, maximum likelihood or maximum-parsimony with Kimura’s two-parameter substitution model (Saitou and Nei 1987). The robustness of the topology of the phylogenetic trees was evaluated by bootstrap analyses based on 1000 resamplings (Galtier et al. 1996; Felsenstein 1985).

### DNA base composition and DNA–DNA hybridization

The G+C content of DNA was measured using the thermal denaturation method described by Marmur and Doty (1962) by using *Escherichia coli* K-12 as the standard. DNA–DNA relatedness (hybridization) was determined by using the spectrophotometric method reported by De Ley et al. (1970).

### Amplification of symbiotic genes and in vivo symbiosis measured in cross-nodulation tests

Symbiotic genes, *nodA* and *nifH*, were amplified by using the primers *nifH*F (5′-TACGGNAARGGSGGNATCGGCAA-3′)/*nifH*I (5′-AGCATGTCYTCSAGYTCNTCCA-3′) and *nodA*F (5′-TGCRGTGGARDCTRYGCTGGGAAA-3′)/*nodA*R (5′-GGNCCGTCRTCRAASGTCARGTA-3′) with PCR conditions previously described by Elliott et al. (2007), Laguerre et al. (2001) and Xu et al. (2013). Formation of symbiosis was also measured in vivo in greenhouse pot experiments by using eight different plant species including the original host plant (Graham et al. 1991): *Sophora alopecuroides*, *Medicago sativa*, *Phaseolus vulgaris*, *Pisum sativum*, *Vigna unguiculata*, *Trifolium repens*, *Glycine max*, *Galega oficinalis* and *N. sibirica*. Briefly, plant seedlings were grown in pots filled with vermiculite moistened with N-free plant nutrient solution (Vincent 1970), inoculated with strain 36-5-1^T^ and formation of nodules determined visually.

### Morphological tests and physiological characterization

The bacterial strain was characterized on the basis of cell morphology including colony color, shape, size, and growth on certain media. To study the cell motility and shape, single colonies isolated from agar plates were prepared by using a JEM-1400, JEDL scanning electron microscope (SEM) and SU8010, Hitachi transmission electron microscope (TEM).

The ability to use common nitrogen and carbon sources, and resistance to common antibiotics, were tested as described by Gao et al. (1994). Additional enzyme activities and biochemical features were determined by using API ZYM, API 20NE kits and API 50 CH at 25 °C as recommended by the manufacturer (bioMérieux). The temperature range and salinity tolerance was investigated by incubating the bacterium on R2A agar at different temperatures (4, 10, 20, 25, 28, 30, 37 and 40 °C) and in medium containing 1, 2, 3, 4, 5, 6, 7, 8% (w/v) of NaCl. The pH range for strain 36-5-1^T^ was measured in R2A broth adjusted with HCl or NaOH to different pH values across the range from 4.0 to 13.0 at intervals of 1.0 pH unit. Dye and chemical resistance were investigated by using methyl orange, methyl red, methylene blue, neutral red, congo red, malachite green, bromothymol blue and sodium deoxycholate at concentrations of 1 and 2% (w/v) in R2A medium.

### Predominant ubiquinone, fatty acid profile and polar lipids

Ubiquinone is an essential component of electron transfer systems in the plasma membrane of prokaryotes, while fatty acid profiling is widely used in the description of rhizobial species (Tighe et al. 2000). To measure these properties, strain 36-5-1^T^ was grown on YMA medium with shaking at 170 rpm for 2 days at 25 °C. Cellular fatty acids were extracted and methylated according to the standard protocol described by Sasser (1990), analyzed by Gas Chromatography (GC) (model 6890; Agilent) and identified by using the TSBA6 database of the Microbial Identification System. Ubiquinone was analyzed by using reversed-phase High Performance Liquid Chromatography (HPLC) and a Diamonsil C18 chromatographic column (200 mm × 4.6 mm, i. d. 5 μm) with 300 ml methanol and 700 ml anhydrous ethanol as the mobile phase. Cellular polar lipids were extracted by using a chloroform–methanol system and separated by two-dimensional TLC using silica gel 60 F 254 aluminium-backed thin-layer plates (Merck) (Kates 1986). The following ratios were used in the solvent system 65:24:4 by volume of chloroform/methanol/water and 80:12:15:4 by volume of chloroform/glacial acetic acid/methanol/water in the first and second dimensions, respectively. Separated components were visualized by treating the plates with a 50% (w/v) sulfuric acid ethanol solution followed by heating at 120 °C for 10 min. Zinzadze reagent was used to detect phospholipids.

### Sequence deposition

The GenBank accession numbers for the 16S rRNA, *glnA* and *atpD* gene sequences of strains 36-5-1^T^ are KU358684, KY523102 and KX095238, respectively.

## Results and discussion

### Phylogenetic analysis based on 16S rRNA, *recA* and *atpD* sequence comparisons

**Fig. 1:**
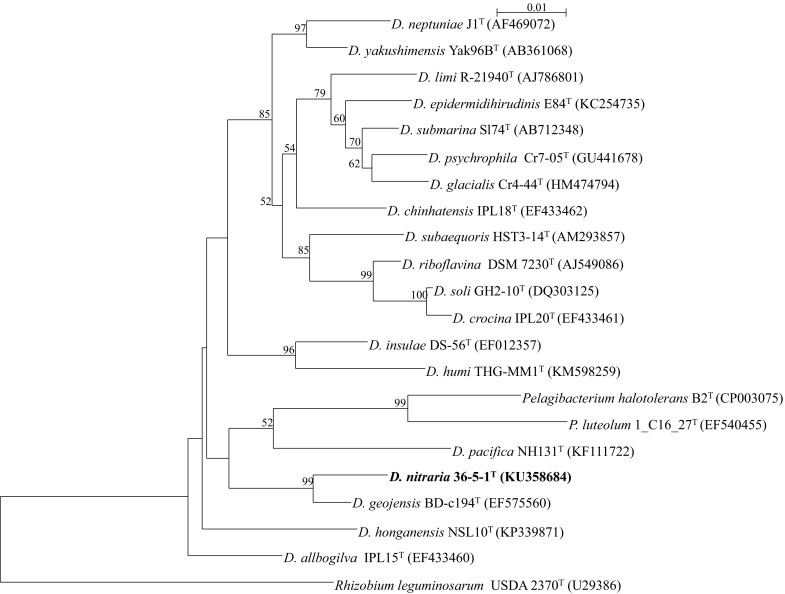
Comparison of 16S rRNA gene sequences. Phylogenetic tree constructed by the NJ method showing the relationships between the novel species and reference strains. Bootstrap percentages above 50% are indicated. *Bar* denotes for 0.1 substitutions per nucleotide position

**Fig. 2:**
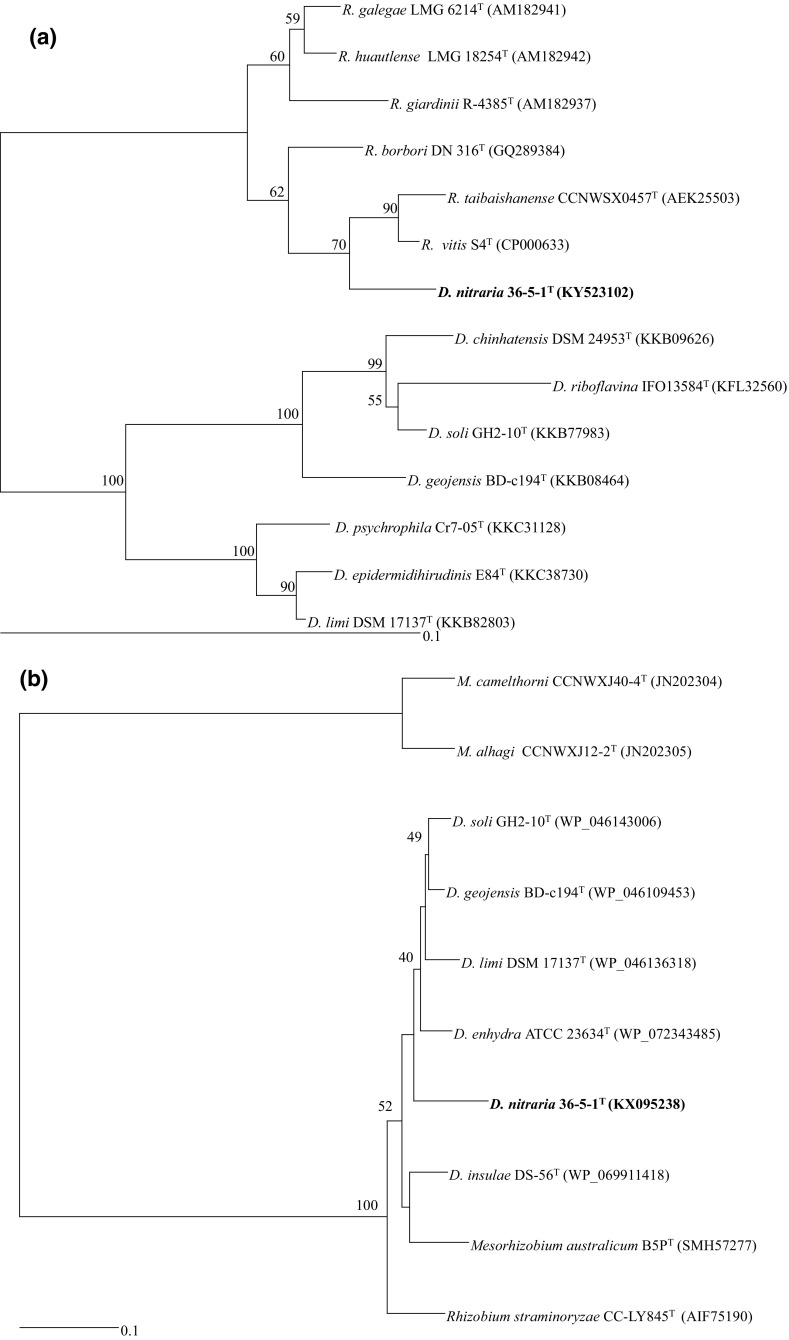
**a** Comparison of partial *gln*A sequences. Phylogenetic tree was constructed by the neighbor-joining method from Jukes–Cantor distance matrices of the sequences. Bootstrap percentages above 50% are indicated. Bar denotes for 0.1 substitutions per nucleotide position. **b** Comparison of partial *atp*D sequences. The phylogenetic tree was constructed by the neighbor-joining method from Jukes–Cantor distance matrices of the sequences. Bootstrap percentages above 50% are indicated. *Bar* denotes for 0.1 substitutions per nucleotide position

Based on the 16S rRNA gene sequence analysis, strain 36-5-1^T^ is phylogenetically highly related to members of the genus *Devosia* and shows close sequence similarity to *Devosia pacifica* NH131^T^ (98.3%) and *Devosia geojensis* BD-c194^T^ (98.6%) (Figs. 1, S1). The phylogenetic trees based on 16S RNA genes indicated that the strain clustered with species of the genus *Devosia*. In addition, the phylogenetic tree based on *glnA* and *atpD* gene sequences indicated that strain 36-5-1^T^ fell into a coherent lineage with species of the genus *Rhizobium* and *Devosia*. The partial *glnA* and *atpD* gene sequences were 96.2 and 91.1% similar to type strains of *Rhizobium vitis* and *Devosia soli*, respectively (Fig. 2). Considering the relatively low sequence similarities of these two genes as a cut-off for genera delineation, this data suggests that strain 36-5-1^T^ belongs to a novel species of the genus *Devosia*.

### DNA–DNA relatedness (hybridization)

The G+C content for the representative strain 36-5-1^T^ was 61.7 mol% (Table 1). Two type strains of closely related species, *D. pacifica* NH131^T^ and *D. geojensis* BD-c194^T^, were used as the reference strains for phenotypic and the hybridization studies. The DNA–DNA relatedness of the strain 36-5-1^T^ with these two closely related species was 44.1 and 40.2%, respectively (Table 1).

**Table 1:** Isolates and reference strains in the genus *Devosia* used in this study and DNA–DNA relatedness among them

Strain	G+C (mol%)	DNA relatedness with 36-5-1^T^ (%)
*D. nitraria* 36-5-1^T^	61.7	100%
Reference strains		
*D. geojensis* BD-c194^T^	60.8	44.1 ± 1.1
*D. pacifica* NH131^T^	63.0	40.2 ± 1.7

### Amplification of symbiotic gene sequences and in vivo symbiosis measured in cross-nodulation tests

Some species of the genus *Devosia* have the ability to form a symbiosis with plants in order to fix nitrogen (Bautista et al. 2010; Rivas et al. 2003). The symbiosis encoding genes are adaptive, and in many cases, have an evolutionary history independent of the rest of the genome. We were not able to obtain PCR products for either *nifH* or *nodA* genes by using the corresponding primers and PCR conditions described previously. Similarly, strain 36-5-1^T^ could not form nodules on the root of any of the tested plants including its original host plant. This suggests that 36-5-1^T^ is not able to nodulate or fix nitrogen for the tested plants. However, considering there are more than 19,000 legume species, strain 36-5-1^T^ may not necessarily have the same symbiotic test results with other legumes.

### Morphological tests and physiological characterization

**Fig. 3:**
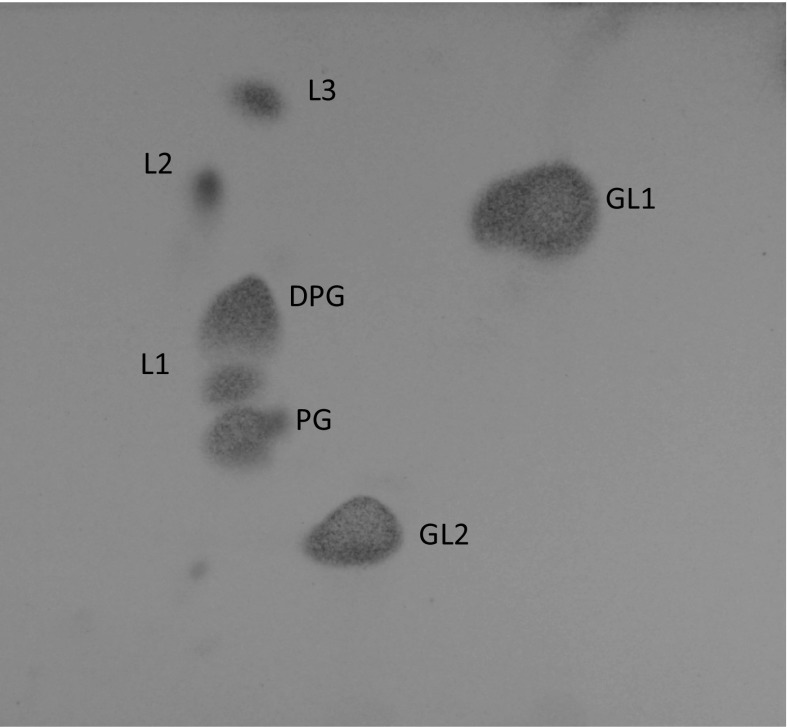
**a** Scanning electron micrograph of strain 36-5-1^T^ grown on YMA media for 48 h. *Bar* indicates 1.0 μm. **b** Transmission electron micrograph of strain 36-5-1^T^ grown on YMA media for 48 h. *Bar* indicates 1.0 μm

Distinctive phenotypic characteristics for the strains of the novel species and the type strains of the phylogenetically closest species are shown in Table 2. An electron micrograph is shown in Fig. 3.

**Table 2:** Distinctive features of *D. nitraria* 36-5-1^T^ and the closely related species, *D. pacifica* and *D. geojensis*

Characteristic	1	2	3
Nitrate reductase	+	**−**	+
Hydrolysis of			
Casein	+	+	**−**
Aesculin	+	+	+
Salicine	+	+	+
Urea	+	+	+
Casei	**−**	**−**	**−**
Tween 80	**−**	**−**	**−**
Gelatin	+	**−**	+
Starch	**−**	**−**	**−**
l-Tyrosine	**−**	+	+
Inuline	+		**−**
*N*-acetyl glucosamine	+	+	+
Phenylacetic acid	**−**	**−**	**−**
Assimilation of			
Fructose	+	+	+
Gentiobiose	+	+	+
Sucrose	**−**	+	+
Asparagine	**−**	+	+
Arginine	+	**−**	+
Erythritol	**−**	**−**	**−**
Sodium glutamate	**−**	+	+
Sodium malate	**−**	+	+
Sodium citrate	**−**	**−**	+
Pyruvic acid sodium	+	+	+
Rhamnose	**−**	+	**−**
Ribose	**−**	+	+
d-Arabinose	+	**−**	**−**
l-Arabinose	+	+	+
Salicin	**−**	+	+
Raffinose	**−**	+	+
d-Trehalose	+	+	+
Succinic acid	+	+	+
d-Galactose	**−**	**−**	**−**
d-Adonitol	+	**−**	**−**
Xylitol	**−**	**−**	**−**
Mannopyranose	**−**	**−**	**−**
l-Homocysteine	+	+	+
l-Alanine	+	+	+
dl-Histidine	+	+	+
l-Glutamic	+	+	+
Methionine	+	+	+
l-Phenylalanine	+	+	+
l-Threonine	+	+	+
l-Leucine	**−**	+	+
Xanthine	+	+	+
Tryptophan	**−**	**−**	**−**
d-Melezitose	+	**−**	**−**
Potassium gluconate	**−**	**−**	**−**
Potassium 2-ketogluconate	**−**	**−**	**–**
Potassium 5-ketogluconate	**−**	**−**	**−**
Acid production from			
d-Xylose	+	+	+
d-Cellobiose	+	+	+
l-Xylose	+	**−**	**−**
d-Glucose	+	+	+
l-Fucose	+	**−**	**−**
d-Fucose	**−**	**−**	**−**
d-Sorbitol	+	+	+
Sorbose	+	**−**	+
Inositol	**−**	+	+
Mannitol	**−**	+	+
Methyl a-d-mannose	+	+	+
*N*-acetyl glucosamine	+	+	+
Amygdalin	+	**−**	**−**
Maltose	+	+	+
Lactose	**−**	+	**−**
d-Tagatose	+	**−**	+
Sucrose	+	+	+
d-Melibiose	**−**	+	+
Resistance to antibiotics			
Polymyxin	**−**	+	+
Streptomycin	**−**	+	**−**
Gentamicin	**−**	+	**−**
Neomycin	+	+	+
Tetracycline	+	**−**	**−**
Chloramphenicol	**−**	+	**−**
Ampicillin	+	**−**	**−**
Kanamycin	**−**	+	**−**
Production of			
Oxidase	+	+	+
Cytochrome oxidase	+	+	+
Catalase	+	+	+

Taxa: 1, *D. pacifica* NH131^T^; 2, *D. geojensis* BD-c194 ^T^; 3. *D. nitraria* 36-5-1^T^. +, positive; −, negative

### Ubiquinone, fatty acid analysis and polar lipids

**Fig. 4:**
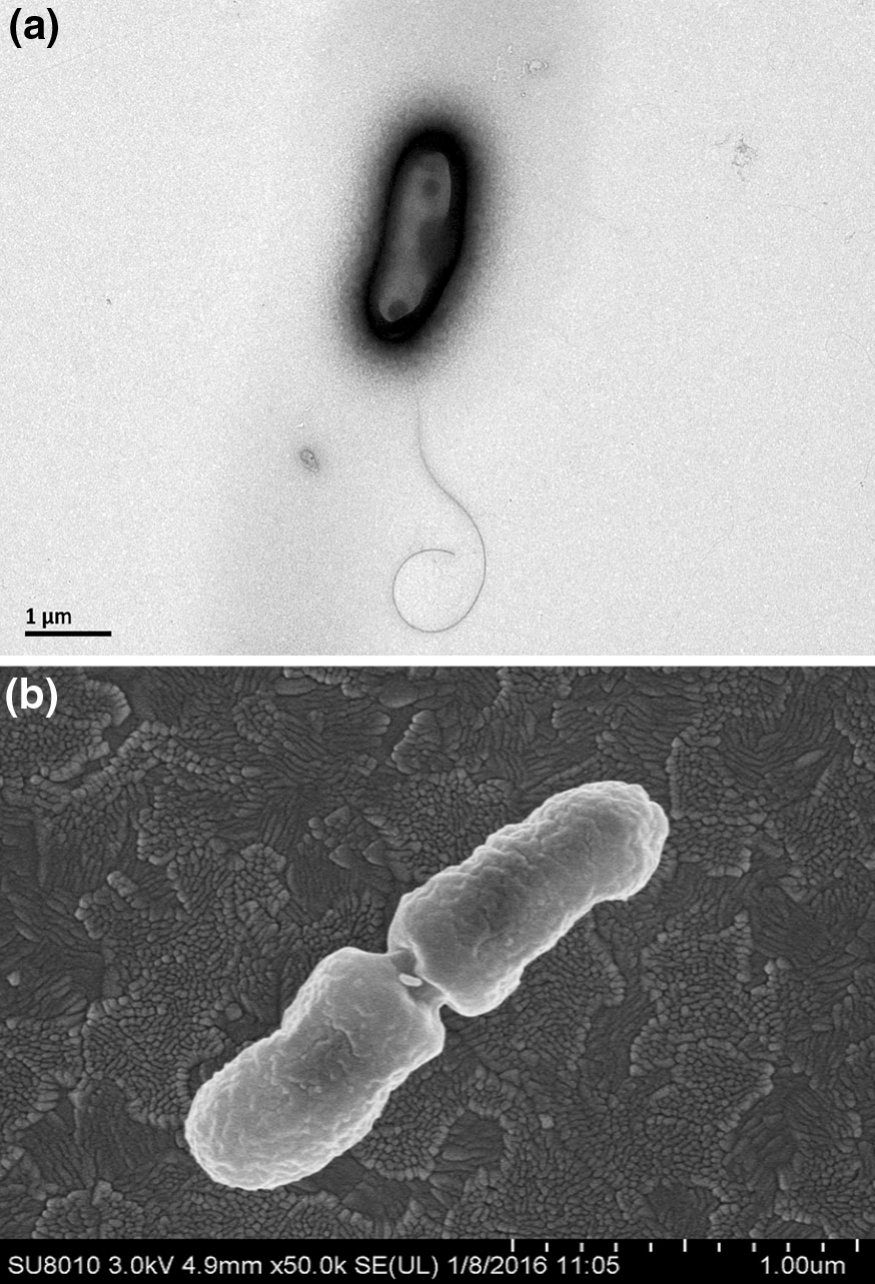
Polar lipid profile of strain 36-5-1^T^ after two-dimensional TLC and staining with molybdatophosphoric acid. *DPG* diphosphatidylglycerol, *PG* phosphatidylglycerol, *GL* unidentified glycolipid L, 1–3 unidentified polar lipids

The predominant ubiquinone of strain 36-5-1^T^ is Q-10. The major fatty acid is C_16:0_ (36.8%) with minor amounts of C_16:0_ N alcohol (14.6%), 10-methyl C_17:0_ (12.6%), C_18:1_ *ω*7*c* (9.4%), C_18:0_ (7.3%), C_19:0_ cyclo ω8*c* (5.5%), 11-Methyl C_18:1_ω7c (4.7%) and C_18:0_ 3-OH (2.5%) (Table 3). The cellular fatty acid profile was similar to the reference strains *D. geojensis* BD-c194^T^ (Ryu et al. 2008) and *D. pacifica* NH131^T^ (Jia et al. 2014) that have C_16:0_ and C_18:1_ *ω*7*c* as their main lipids, respectively. A large amount of unidentified glycolipid, diphosphatidylglycerol, phosphatidylglycerol and a small amount of unidentified polar lipids were present as polar lipids for strain 36-5-1^T^ (Fig. 4).

**Table 3:** Cellular fatty acids of strain 36-5-1^T^

Fatty acid	*D. nitraria* 36-5-1^T^
C_14:0_	1.6
C_15:0_	–
C_16:0_	36.8
C_17:0_	0.6
C_18:0_	7.3
C_19:0_	–
C_16:0_ N alcohol	14.6
C_17:0_ cyclo	0.7
C_16:1_ *ω*7*c*/_16:1_ *ω*6*c*	1.9
C_17:1_ *ω*8*c*	–
C_17:1_ *ω*6*c*	–
C_18:1_ *ω*5*c*	–
C_18:1_ *ω*7*c*	9.4
C_18:1_ *ω*9*c*	–
C_20:1_ *ω*7*c*	–
C_18:3_ *ω*6c (6,9,12)	1.3
C_8:0_ 3-OH	–
C_18:0_ 3-OH	2.5
10-Methyl C_17:0_	12.6
11-Methyl C_18:1_ω7c	4.7
C_19:0_ cyclo ω8*c*	5.5
ECL18.814	–

### Description of *Devosia nitraria* sp. nov.

*Devosia nitraria* (Ni.tra’ri. N.L. gen. n. *nitraria*, referring to the host plant *Nitraria sibirica*).

 Gram-negative, aerobic, motile (flagella), non-spore forming rod, which is 0.3–0.5 μm wide and 1.0–1.5 μm long. Colonies on R2A medium are circular, convex, white and semitranslucent, with a typical diameter of 3–5 mm after 3 days of growth at 25 °C. Cells are positive for utilization of inositol, l-arabinose, d-mannitol, d-sorbitol, gentiobiose, glucose, sodium citrate, sodium malonate, pyruvic acid sodium, sucrose, d-xylose, d-ribose, d-fructose, d-mannose, d-maltose, sodium acetate, esculine, salicine, amygdaline, d-melibiose, d-saccharose, d-trehalose, d-raffinose, and d-tyranose as sole carbon sources but negative for d-galactose, l-rhamnose, erythritol, d-arabinose, d-adonitol, l-xylose, l-sorbose, inuline, dulcitol, xylitol, l-fucose, d-fucose, d-arabitol, d-lactose and mannopyranose as the sole carbon sources. Cells are positive for utilization of l-homocysteine, l-alanine, dl-histidine, l-tyrosine, l-leucine, l-threonine, methionine, l-phenylalanine, l-arginine, l-glutamic, aspartic acid and xanthine but negative for tryptophan as a sole nitrogen source. Optimum growth occurs at 30 °C and the strain’s growth is inhibited at temperature extremes of 4 and 40 °C in R2A medium, as well as at 37 and 40 °C when grown in LB and YMA media, respectively. Optimum pH for growth is at 7.0 while some growth is observed up to pH levels of 11.0. Cells grow on YMA in the presence of 7% (w/v) NaCl but do not grow in media supplemented with 0.1% malachite green, 0.1% methylene blue and 0.1% neutral red. Cells are sensitive to 10 μg ml^−l^ tetracycline, gentamicin, chloramphenicol, ampicillin, streptomycin and kanamycin, but resistant to polymyxin and neomycin. Cells are negative for casein hydrolysis, litmus milk alkali production, nitrate reduction, Voges-Proskauer, d-melezitose, potassium gluconate, potassium 2-ketogluconate and potassium 5-ketogluconate, but positive for d-cellobiose, urease, cytochrome oxidase, catalase oxidase and hydrolysis of gelatin. Cells can assimilate *N*-acetyl glucosamine but not phenylacetic acid. The predominant ubiquinone is Q-10. The main cellular fatty acid is C_16:0_.

The type strain, 36-5-1^T^ (=CGMCC1.15704^T^=NBRC112416^T^), was isolated from the nodules of *Nitraria sibirica* grown in Hexi corridor in Zhangye city, Gansu province of China.

## Electronic supplementary material

Below is the link to the electronic supplementary material.

**Fig. S1** (a) Maximum Likelihood tree reconstructed from 16S rRNA gene sequences of 36-5-1^T^ and related type strains. Bootstrap values (based on 1000 replicates) above 50 % are indicated at the nodes. Bar denotes for 0.1 substitutions per nucleotide position. (b) Maximum Parsimony tree reconstructed from 16S rRNA gene sequences of 36-5-1^T^ and related type strains. Bootstrap values (based on 1000 replicates) above 50 % are indicated at the nodes (PPTX 87 kb)
