# Supplementary material for: Devosia nitraria sp. nov., a novel species isolated from the roots of Nitraria sibirica in China
Source: Antonie Van Leeuwenhoek. 2017 Jun 24;110(11):1475–83. doi: 10.1007/s10482-017-0901-z (PMC5644702; doi:10.1007/s10482-017-0901-z)
Supplement: Supplementary file 1 — Fig. S1 (a) Maximum Likelihood tree reconstructed from 16S rRNA gene sequences of 36-5-1T and related type strains. Bootstrap values (based on 1000 replicates) above 50 % are indicated at the nodes. Bar denotes for 0.1 substitutions per nucleotide position. (b) Maximum Parsimony tree reconstructed from 16S rRNA gene sequences of 36-5-1T and related type strains. Bootstrap values (based on 1000 replicates) above 50 % are indicated at the nodes (PPTX 87 kb) [file 10482_2017_901_MOESM1_ESM.pptx]

## Slide 1
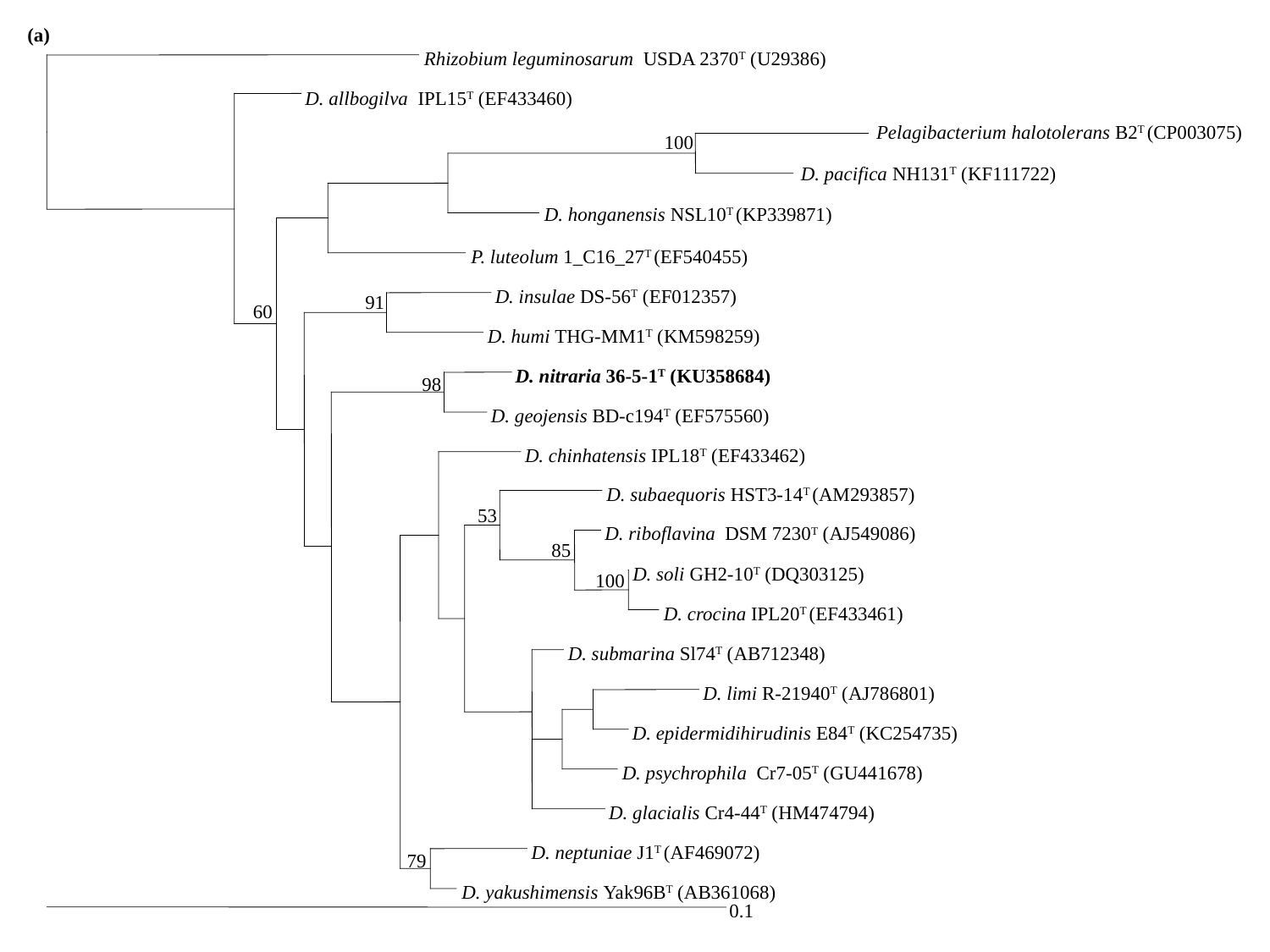

(a)
Rhizobium leguminosarum USDA 2370T (U29386)
D. allbogilva IPL15T (EF433460)
Pelagibacterium halotolerans B2T (CP003075)
100
D. pacifica NH131T (KF111722)
D. honganensis NSL10T (KP339871)
P. luteolum 1_C16_27T (EF540455)
D. insulae DS-56T (EF012357)
91
60
D. humi THG-MM1T (KM598259)
D. nitraria 36-5-1T (KU358684)
98
D. geojensis BD-c194T (EF575560)
D. chinhatensis IPL18T (EF433462)
D. subaequoris HST3-14T (AM293857)
53
D. riboflavina DSM 7230T (AJ549086)
85
D. soli GH2-10T (DQ303125)
100
D. crocina IPL20T (EF433461)
D. submarina Sl74T (AB712348)
D. limi R-21940T (AJ786801)
D. epidermidihirudinis E84T (KC254735)
D. psychrophila Cr7-05T (GU441678)
D. glacialis Cr4-44T (HM474794)
D. neptuniae J1T (AF469072)
79
D. yakushimensis Yak96BT (AB361068)
0.1

## Slide 2
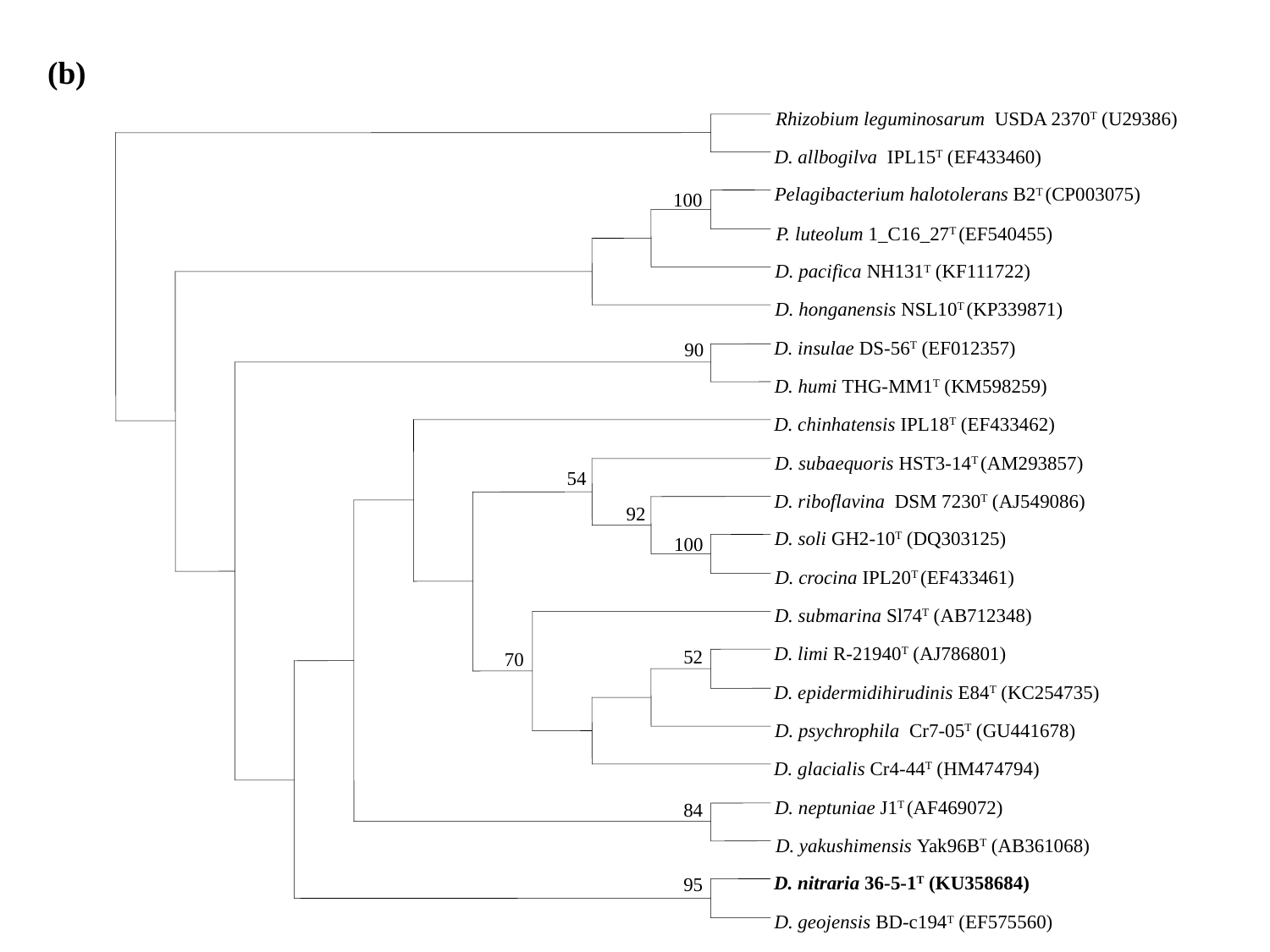

(b)
Rhizobium leguminosarum USDA 2370T (U29386)
D. allbogilva IPL15T (EF433460)
Pelagibacterium halotolerans B2T (CP003075)
100
P. luteolum 1_C16_27T (EF540455)
D. pacifica NH131T (KF111722)
D. honganensis NSL10T (KP339871)
D. insulae DS-56T (EF012357)
90
D. humi THG-MM1T (KM598259)
D. chinhatensis IPL18T (EF433462)
D. subaequoris HST3-14T (AM293857)
54
D. riboflavina DSM 7230T (AJ549086)
92
D. soli GH2-10T (DQ303125)
100
D. crocina IPL20T (EF433461)
D. submarina Sl74T (AB712348)
D. limi R-21940T (AJ786801)
52
70
D. epidermidihirudinis E84T (KC254735)
D. psychrophila Cr7-05T (GU441678)
D. glacialis Cr4-44T (HM474794)
D. neptuniae J1T (AF469072)
84
D. yakushimensis Yak96BT (AB361068)
D. nitraria 36-5-1T (KU358684)
95
D. geojensis BD-c194T (EF575560)
